# Targeted in-vitro-stimulation reveals highly proliferative multi-virus-specific human central memory T cells as candidates for prophylactic T cell therapy

**DOI:** 10.1371/journal.pone.0223258

**Published:** 2019-09-30

**Authors:** Benjamin Faist, Fabian Schlott, Christian Stemberger, Kevin M. Dennehy, Angela Krackhardt, Mareike Verbeek, Götz U. Grigoleit, Matthias Schiemann, Dieter Hoffmann, Andrea Dick, Klaus Martin, Martin Hildebrandt, Dirk H. Busch, Michael Neuenhahn

**Affiliations:** 1 Institute for Medical Microbiology, Immunology and Hygiene, Technische Universität München, Munich, Germany; 2 German Center for Infection Research (DZIF), partner site Munich, Munich, Germany; 3 Juno Therapeutics, Munich, Germany; 4 German Center for Infection Research (DZIF), partner site Tübingen, Tübingen, Germany; 5 Institute for Medical Virology, University Hospital Tübingen, Tübingen, Germany; 6 Department of Medicine III, Klinikum Rechts der Isar, Technische Universität München, Munich, Germany; 7 Department of Internal Medicine II, University of Würzburg, Wuerzburg, Germany; 8 Institute for Virology, Technische Universität München, Munich, Germany; 9 Department of Transfusion Medicine and Haemostaseology, Ludwig-Maximilians-Universität München, Munich, Germany; 10 Institute of Anaesthesiology, Deutsches Herzzentrum München, Klinik an der Technischen Universität München, Munich, Germany; 11 TUM Cells Interdisciplinary Center for Cellular Therapies, Munich, Germany; University of St Andrews, UNITED KINGDOM

## Abstract

Adoptive T cell therapy (ACT) has become a treatment option for viral reactivations in patients undergoing allogeneic hematopoietic stem cell transplantation (alloHSCT). Animal models have shown that pathogen-specific central memory T cells (T_CM_) are protective even at low numbers and show long-term survival, extensive proliferation and high plasticity after adoptive transfer. Concomitantly, our own recent clinical data demonstrate that minimal doses of purified (not in-vitro- expanded) human CMV epitope-specific T cells can be sufficient to clear viremia. However, it remains to be determined if human virus-specific T_CM_ show the same promising features for ACT as their murine counterparts. Using a peptide specific proliferation assay (PSPA) we studied the human Adenovirus- (AdV), Cytomegalovirus- (CMV) and Epstein-Barr virus- (EBV) specific T_CM_ repertoires and determined their functional and proliferative capacities *in vitro*. T_CM_ products were generated from buffy coats, as well as from non-mobilized and mobilized apheresis products either by flow cytometry-based cell sorting or magnetic cell enrichment using reversible Fab-Streptamers. Adjusted to virus serology and human leukocyte antigen (HLA)-typing, donor samples were analyzed with MHC multimer- and intracellular cytokine staining (ICS) before and after PSPA. T_CM_ cultures showed strong proliferation of a plethora of functional virus-specific T cells. Using PSPA, we could unveil tiniest virus epitope-specific T_CM_ populations, which had remained undetectable in conventional *ex-vivo*-staining. Furthermore, we could confirm these characteristics for mobilized apheresis- and GMP-grade Fab-Streptamer-purified T_CM_ products. Consequently, we conclude that T_CM_ bare high potential for prophylactic low-dose ACT. In addition, use of Fab-Streptamer-purified T_CM_ allows circumventing regulatory restrictions typically found in conventional ACT product generation. These GMP-compatible T_CM_ can now be used as a broad-spectrum antiviral T cell prophylaxis in alloHSCT patients and PSPA is going to be an indispensable tool for advanced T_CM_ characterization during concomitant immune monitoring.

## Introduction

The number of performed allogeneic hematopoietic stem cell transplantations (alloHSCTs) is continuously rising and myeloid malignancies are their major indication [1]. However, despite improvements over the last decades mortality after alloHSCT still remains a major challenge. Beside relapse of the myeloid malignancy intervention-associated risk factors like graft versus host disease (GVHD) and opportunistic infections are the leading causes of fatal outcomes following alloHSCT [2] [3]. Co-transferred T cells within the stem cell graft are a double edged-sword. They play a crucial role in the prevention of opportunistic (especially viral) infections and can mediate GVL (graft versus leukemia) effects, but they can simultaneously also lead to GVHD [3]. Currently, there are only few licensed antiviral drugs available, which are limited by spectrum–lacking significant efficacy against Adenovirus (AdV) and EBV (Epstein-Barr virus)–or do not lead to sufficient clinical improvement [4,5]. Furthermore, side effects [6–8] and drug resistances [9,10] further limit their use. Letermovir is a recently approved antiviral drug that seems to be an attractive candidate for prevention and potentially also for treatment of CMV [11]. However, it lacks activity against other viruses and there are currently no data that promise a dramatic change regarding this situation in the near future [12–14]. In contrast, it has been shown in several studies that control of virus infections is dependent on T cell immunity. Adoptive transfer of T cells has shown encouraging results in several clinical studies [15–25]. However, the T cell products that are used by adoptive T cell therapy (ACT)-performing centers are highly diverse. In-vitro-expansion of T cells is a well-established method, in which lysates of infected cells or viral protein-spanning synthetic peptides can be used for sensitization of T cells against viruses of choice but, beside high costs, it is very time-consuming until a therapeutic product is generated and ready for transfusion. In contrast, T cell products generated via cytokine capture methods, which are usually CD4^+^ T cell-biased [18], can be generated by short term sensitization and are already available within 24–48 hours. However, both methods underlie in Europe strict ATMP (Advanced Therapy Medicinal Products)-standards, which can be high regulatory barriers for most centers. Furthermore, activation of T cells by in-vitro-stimulation could possibly influence their functionality, proliferative and survival capacity [26]. With the availability of reversible MHC-Streptamers, minimally manipulated (“quasi untouched”) GMP (Good manufacturing practice)-grade antigen-specific CD8^+^ T cells can be isolated directly *ex vivo*. These highly purified T cell products simultaneously fulfill timely availability and meet important regulatory requirements [27,28]. Recently, several clinical data have supported the potential of MHC-Streptamer reagents for the isolation of stem cell-donor-derived virus-specific T cells [19,29,30]. However, this method requires the availability of MHC/HLA-restricted virus-specific reagents matching to at least one of the patient’s HLA molecules and depends on the presence of the targeted T cell population in a size that enables sufficient ex-vivo-isolation. Therefore, MHC-Streptamer selection can reach its limits for small T cell populations, rare HLA-types or opportunistic pathogens for which only limited knowledge about immunodominant epitopes exists. Furthermore, due to the lack of GMP-compliant MHC-II-Streptamers pathogen-specific CD4^+^ T cells cannot be enriched with this method.

In summary, adoptive transfer of T cells addresses an important clinical need and is strikingly efficient and safe, but it can benefit from further refinement. Accordingly, recent findings are driving the focus for optimal ACT products towards the use of T cell subsets with a specific differentiation profile rather than considering exclusively antigen specificity [31]. T_CM_ as early-differentiated memory T cells have been described to possess several promising features in this context. High differentiation and proliferation capacity of minimal numbers of T_CM_ as well as long-term persistence and protection have been found in murine and primate models [19,32–35]. At the same time and in contrast to naïve T cells, T_CM_ show a rather beneficial GVHD profile [36,37]. Furthermore, recently developed Fab-Streptamers allow the clinical-grade ex-vivo-isolation of minimally manipulated T_CM_, in similarity to the established MHC-Streptamer technology [38].

Based on these observations, we considered T_CM_ as interesting candidate cells for prophylactic broad-spectrum ACT and analyzed human T_CM_ of different donor origins within this manuscript. We focused on the capacities of human virus-specific T_CM_ and observed similar features as described earlier in animal models. Functional T_CM_-derived progenies proliferated extensively and revealed a diverse spectrum of virus-specific T cell specificities, which identifies T_CM_ as an attractive compartment for prophylactic T cell therapy.

## Materials and methods

### Donor material, PBMC isolation and cryopreservation

T cell subset analysis of CMV-specific T cells was effectuated with peripheral blood mononuclear cells (PBMC) from healthy T cell donors [29]. PBMC were isolated from fresh donor material and stained for flow cytometry. Written informed consent was obtained from all donors and usage of the blood samples was approved by the responsible Institutional Review Board (Ethics committee of the Faculty of Medicine, University Würzburg (11/08)) covering the presented work reported in this manuscript.

Donor material for T_CM_ isolation was collected from healthy (male and female) individuals collected at the Faculty of Medicine, Technische Universität München, Munich, Germany. Samples originated either from buffy coats gained from autologous blood donors or from non-mobilized leukapheresis material that was generated in a volunteer setting. Peripheral blood mononuclear cells (PBMC) were isolated from fresh donor material by density gradient centrifugation as described earlier [39]. Afterwards, PBMC samples were cryopreserved in liquid nitrogen using a 90% fetal calf serum (FCS, Biochrom GmbH, Berlin, Germany) and 10% dimethyl sulfoxide (DMSO, Sigma-Aldrich, Taufkirchen, Germany) solution. One additional sample originated from cryopreserved mobilized stem cell apheresis material. Here, the sample was thawed and PBMC isolation was performed afterwards. Written informed consent was obtained from all donors and usage of the blood samples was approved by the local Institutional Review Board according to national law and the declaration of Helsinki and Istanbul (Ethics committee of the Faculty of Medicine, Technical University Munich (360/13 and 55/14) covering the presented work reported in this manuscript. One research apheresis product for exhaustion cell marker analysis of Fab Streptamer-isolated T_CM_ was obtained from Cellex, Dresden under the ethical quote EK309072016 (Ethical committee of the Technical University Dresden) for development of new generation cellular therapies.

### Virus serology and HLA-typing

Before characterization of virus-specific donor T cells, donor IgG serology was determined for Adenovirus (AdV), CMV and EBV. For AdV serology, IgG ELISA (IBL International, Hamburg, Germany) or Novagnost Adenovirus IgG ELISA (Siemens Healthcare Diagnostics, Marburg, Germany) measured on a BEP III System (Siemens Healthcare Diagnostics) was used. For CMV serology, CMV-IgG-ELA Test PCS (Medac, Wedel, Germany) or Architect c4000 (Abbott GmbH & Co. KG, Wiesbaden, Germany) and CMV-IgG reagents (Abbott GmbH & Co. KG) were used. For EBV serology, Enzygnost Anti-EBV IgG (Siemens Healthcare Diagnostics) measured on a BEP III System was used. For leukapheresis volunteers only, molecular HLA-typing was performed by the Laboratory for Immunogenetics and Molecular Diagnostics, Ludwig-Maximilians-Universität München (LMU), Munich, Germany.

### Intracellular cytokine staining and multimer staining

PBMCs were analyzed either *ex vivo*, after cryopreservation or following the peptide-specific in-vitro-culture. Cryopreserved material was thawed by adding the cell suspension to a 50ml Falcon tube with RPMI + 10% FCS (ratio 1:3) together with 0.1mg/ml of DNAse I (F. Hoffmann-La Roche AG, Basel, Switzerland). Cells were centrifuged afterwards and a resuspension step was repeated before resting for 18 hours in RPMI + 10% FCS (2x10^6^ cells/ml) before analysis. For ICS, PBMCs were stimulated with 2¼g/ml of single peptides (generated by IBA GmbH, Göttingen, Germany) or peptide pools (15mer with 11aa overlap spanning the entire protein) in the presence of 1¼g/ml anti-CD28 (BD Biosciences, San Jose, USA) and 1¼g/ml anti-CD49d (BD Biosciences) costimulatory antibodies for 1 hour at 37°/ 5% CO_2_ [39]. For stimulation of AdV-specific T cells we used HLA-A*01:01-restricted LTDLGQNLLY or TDLGQNLLY, HLA-A*24:02-restricted TYFSLNNKF, HLA-B*07:02-restricted KPYSGTAYNAL [40] and HLA-C*07:02-restricted FRKDVNMVL [41] as Hexon-based single peptides and adenovirus serotype 3 hexon protein (PepMix^™^ HAdV-3, JPT Peptide Technologies GmbH, Berlin, Germany), serotype 5 hexon protein (PepTivator AdV5 Hexon, Miltenyi Biotec GmbH, Bergisch-Gladbach, Germany) and serotype 5 penton protein (PepMix™ HAdV-5, JPT) as peptide pools. For stimulation of CMV specific T cell we used HLA-A*02:01-restricted NLVPMVATV, HLA-A*24:02-restricted QYDPVAALF and HLA-B*07:02-restricted TPRVTGGGAM (all pp65 based) and HLA-C*07:02-restricted CRVLCCYVL [35] (IE-1 based) as single peptides and immediate-early protein 1 (IE-1) or the whole 65 kDa phosphoprotein (PepMix™ HCMVA (IE-1) and (pp65), JPT) as peptide pools (all single peptides derived from IBA Lifesciences, Göttingen, Germany). For stimulation of EBV specific T cells we used HLA-A*02:01-restricted GLCTLVAML (BMLF1) as single peptide and BZLF1, EBNA1, EBNA3a, EBNA3c, LMP1 or LMP2 (PepMix™ EBV, JPT) peptide pools. Afterwards 0.01¼g/¼l Brefeldin A (Sigma-Aldrich) was added and incubated for 3.5 hours. For live dead discrimination, cells were stained for 10 minutes on ice with 2¼g/ml ethidium bromide monoazide (EMA, Sigma-Aldrich). Surface staining was performed for 30 minutes on ice using anti-CD3 APC (BD Biosciences, San Jose, USA), anti-CD3 PE-Cy7 (eBioscience, San Diego, USA) or anti-CD3 Alexa700 (BD Biosciences), anti-CD4 v500 (BD Biosciences), anti-CD8 PerCP (BD Biosciences), anti-CD45RO PE-Cy7 (BD Biosciences) or anti-CD45RO PE (Beckmann Coulter, Brea, USA), anti-CD45RA (BD Biosciences) and anti-CD62L eF450 (eBioscience). A separate staining for anti-CCR7 FITC (R&D Minneapolis, USA) was conducted at 37°C to stabilize its surface expression [42]. Afterwards, cells were permeabilized/fixed for 20 minutes on ice using BD^TM^ Cytofix/Cytoperm kit (BD Biosciences) followed by an incubation with IFNγ Alexa Fluor^®^ 700 (eBioscience) for 30 minutes on ice. Cells were measured using a BD^TM^ LSR II (BD Biosciences) and analyzed by FlowJo (FlowJo, LLC, Ashland, USA) software. The frequency of AdV- or CMV-specific CD8^+^ T cells was also determined by staining with MHC-class I reversible multimers, so called Streptamers, composed of a matching HLA Class I molecule bound to the above mentioned AdV- or CMV single peptide epitopes (as described in [27,43]). MHC-monomers were multimerized with either an APC or PE Streptactin (IBA Lifesciences). Following EMA and multimer staining, cells underwent cell surface staining (without permeabilization/fixation) as described above followed by analysis on the LSR II flow cytometer. Staining of HLA-C*07:02-restricted multimers was always accompanied by counterstaining using MAGE-A12_170-178_ (VRIGHLYIL) bound multimer [44,45]. Surface staining for coinhibitory markers (30 minutes on ice) was performed using anti-PD-1 APC (eBioscience, San Diego, USA), anti-LAG-3 FITC (eBioscience, San Diego, USA) and anti TIM-3 PB (eBioscience, San Diego, USA).

### Enrichment of central memory T cells using fluorescence-activated cell sorting

Buffy coat-derived fresh donor PBMC first underwent magnetic bead enrichment of CD3^+^ T cells using reversible CD3 Fab-Streptamers (IBA Lifesciences) as previously described [38]. Following surface staining (see above), unfixed CD3^+^ CD45RO^+^ CD62L^+^ or CD3^+^ CD45RO^+^ CCR7^+^ T_CM_ were sorted on a MoFlo II cell sorter (Beckman Coulter). PBMC from cryopreserved, mobilized stem cell apheresis material were isolated entirely by fluorescence-activated cell sorting. Purity controls revealed purities > 90% for sorted T_CM_.

### Enrichment of central memory T cells using reversible multimer technology

T_CM_ from non-mobilized leukapheresis material of healthy volunteers were isolated in a GMP conform manner at TUMCells (Faculty of Medicine, Technische Universität München, Munich, Germany). Cells were purified in a serial positive enrichment process using a modified method upon expression of CD3, CD62L followed by a depletion of CD45RA using Fab-Streptamer technology [38].

### Peptide specific in-vitro-culture for T cell proliferation

Fresh or cryopreserved donor PBMC were used for autologous stimulation. Cryopreserved PBMC were thawed (with additional DNAse treatment for the mobilized apheresis material) and a resting procedure as described above was initiated. PBMC were transferred in 50ml Falcon tubes, washed twice with RPMI (Sigma-Aldrich), centrifuged at 700g for 5 minutes and counted afterwards. 4x10^6^ cells/ml were labeled with 1μg of the appropriate peptide or peptide mix and incubated for 2h at 37°C and 5% CO_2_. Afterwards the cells were washed twice with RPMI (Sigma-Aldrich) and resuspended in 1ml cell culture medium (RPMI supplemented with 10% FCS, 100 U/ml penicillin and 100 μg/ml streptomycin (both Life Technologies)) followed by irradiation at 35Gy on ice. Cells were counted and 1.25x10^6^ of the peptide-loaded irradiated autologous stimulator PBMCs were seeded together with 0.25x10^6^ viable T_CM_ in a 12-well plate (BD Biosciences) in a volume of 3ml of cell culture medium. Incubation with unloaded irradiated PBMCs served as a negative control and PBMCs stimulated with 3μl/ml anti-CD3 (Becton-Dickinson) of a 1:100 dilution and 0.65μl/ml of anti-CD28 (BD Biosciences) as a positive control. IL-2 (Miltenyi Biotec) was added in a concentration of 50 I.U./ml every 3 days starting on day 3. Change of culture medium was performed every six days and always according to optical/microscopical evaluation depending on medium color and cell density. Cells were restimulated every 10–12 days by adding 1.25x10^6^ of the appropriate peptide-loaded, irradiated stimulator PBMCs. Harvesting of cells was performed not earlier than 10 days after last addition of peptide-loaded, irradiated stimulator PBMCs.

## Results

### Human CMV-specific CD8^+^ T cells with a T_CM_ phenotype occur at low frequencies in peripheral blood

**Fig 1:**
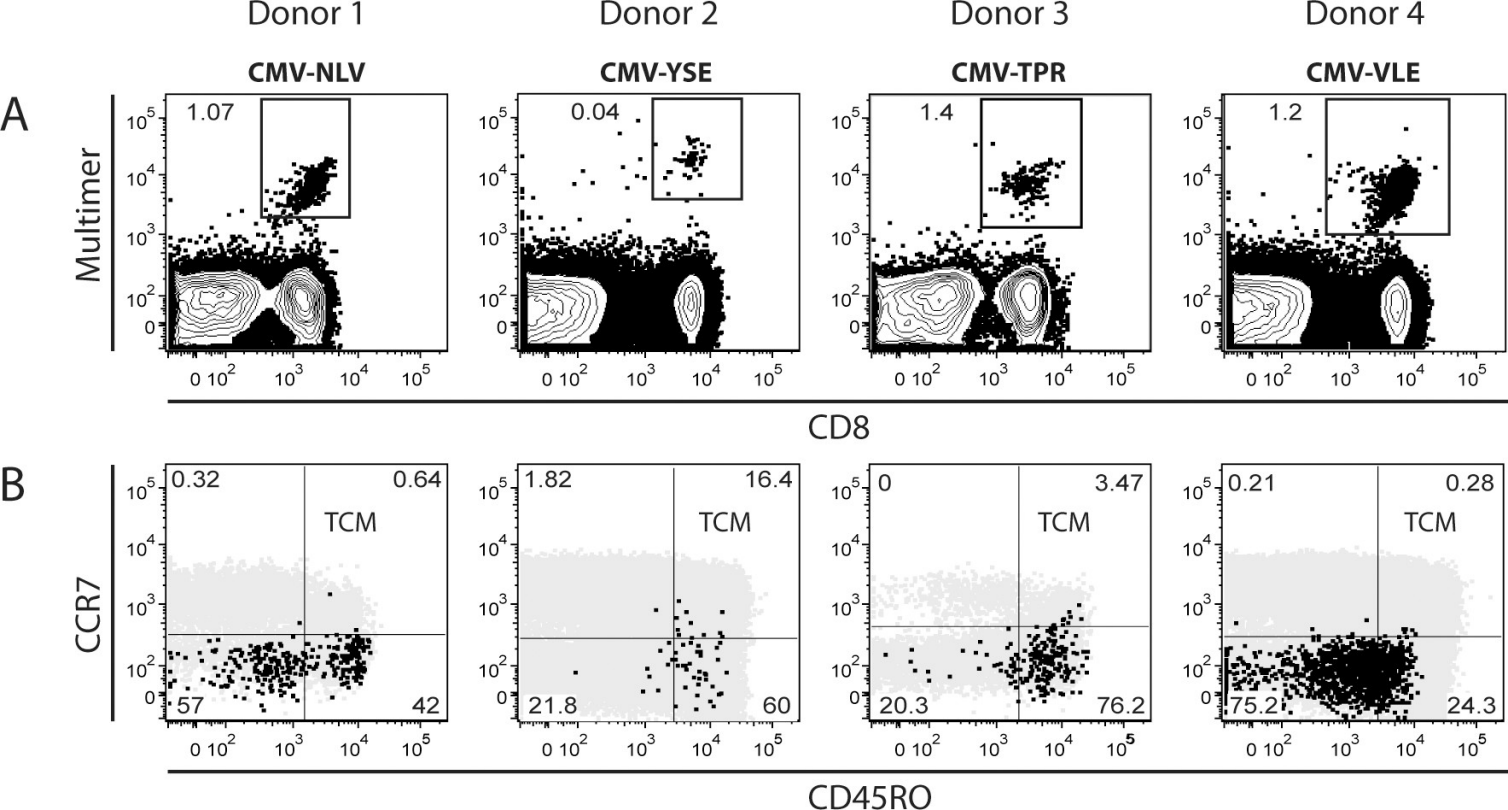
CMV-specific T cells with T_CM_ phenotype are rarely detectable in peripheral blood. Four representative adoptive CMV-ACT donors are depicted. **(A)** CMV epitope-specific CD8^+^ T cells were quantified by staining with CMV pp65-based HLA-A*02:01/NLV- (donor 1), HLA-A*01:01/YSE- (donor 2), HLA-B*07:02/TPR (donor 3) and CMV IE-1-based HLA-A*02:01/VLE (donor 4) MHC-multimers, respectively. **(B)** Phenotypic characterization of corresponding MHC-multimer-positive populations (black) was performed according to expression of CCR7 and CD45RO. Phenotypes were defined as T_N_ (naive T cells, CCR7^+^), T_CM_ (central memory T cells, CD45RO^+^/CCR7^+^), T_EM_ (effector memory T cells, CD45RO^+^/CCR7^-^) and T_EMRA_ (effector T cells, CD45RO^-^/CCR7^-^). Phenotypic distribution of the corresponding CD3^+^ T cell populations (grey) is shown.

Preclinical murine models have shown that minimal numbers of T_CM_ are capable of extensive proliferation, differentiation as well as long-term persistence and protection against intracellular pathogens [19,32–35]. The goal of our study was to examine if human virus-specific T_CM_ show similar promising features and could become candidates for prophylactic broad-spectrum ACT. However, according to previous findings [46] and our own experiences, particularly CMV-specific T cells are dominated by a late-differentiated phenotype in peripheral blood. Four representative healthy donors are shown in Fig 1, where we determined in ex-vivo-isolated PBMC the frequency and phenotype of epitope-specific CD8^+^ T cell populations by CMV-restricted MHC-multimers (Fig 1A). CCR7 and CD45RO served for discrimination of CMV-specific T_N_, T_CM_, T_EM_ and T_EMRA_ phenotypes (Fig 1B). Irrespective of the size, the HLA restriction or the recognized CMV-epitope of the MHC-multimer^+^ T cell population, CMV-specific CD8^+^ T cells with a T_CM_ phenotype are markedly underrepresented in comparison to their T_EM_ or T_EMRA_ counterparts. Donor samples with a higher frequency (> 1.0%) of CMV-specific T cells show only a minimal- (donor 3) or even hardly detectable (donor 1 and 4) CMV-specific proportion of T_CM_ phenotype. Donor 2 contains an overall small CMV-specific T cell population (0.04%) with a minority displaying a T_CM_ phenotype. Furthermore, the frequency of CMV-specific T_CM_ was especially low in donors showing a high proportion of CMV-specific CD8^+^ T cells of the late differentiated T_EMRA_ phenotype (donor 1 and 4). We conclude that the ex-vivo-analysis of circulating CMV-specific T_CM_ is difficult due to the low cell numbers detected, even in donors with large, dominating CMV-specific T cell populations.

### Prolonged peptide-specific stimulation unveils potent features of CMV-specific T cells within the T_CM_ compartment

**Fig 2:**
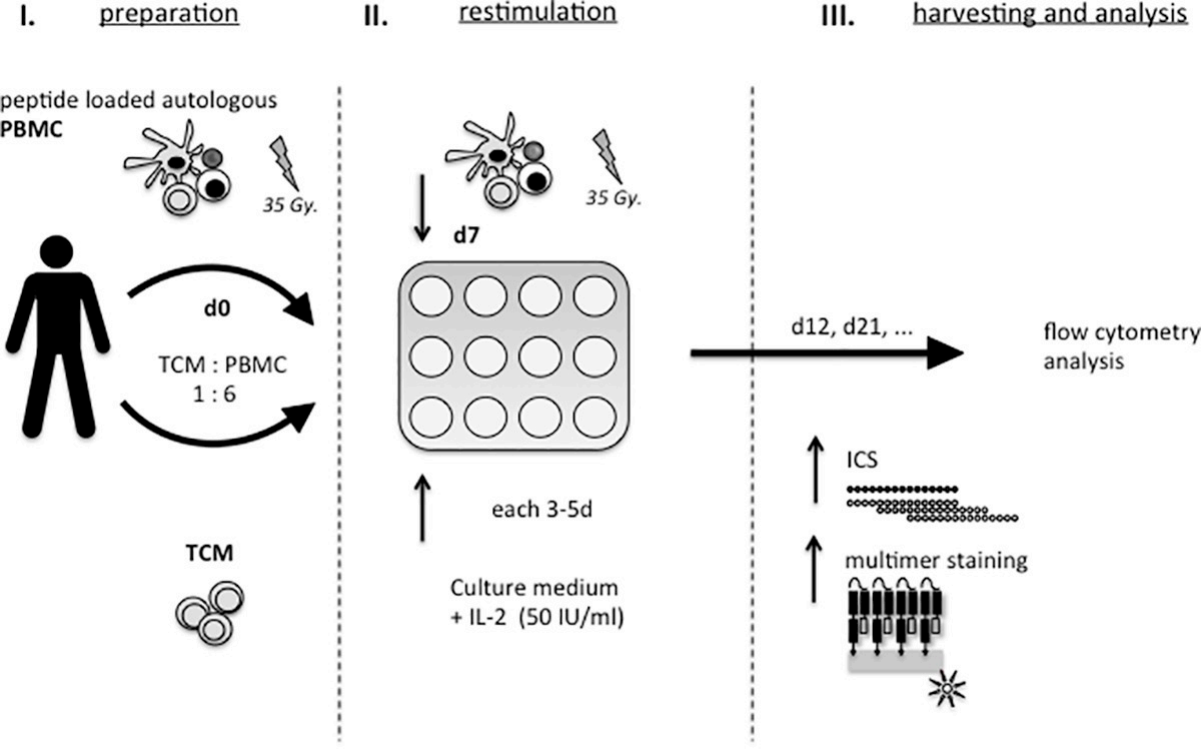
Peptide-specific proliferation assay (PSPA) for detailed analysis of antigen-specific T_CM_. **(I)** T_CM_ are co-cultured with gamma-irradiated peptide-labeled autologous PBMC (1:6 ratio) in a flat-bottom well plate on day 0. **(II)** Cells can be restimulated with corresponding stimulator PBMC on day 7. IL-2 (50 IU/ml) is added at day 3 and thereafter every 3–5 days IL-2-containing medium is used for culture medium exchange. **(III)** Cells can be primarily harvested on day 12–21. After harvest, they either undergo stimulation with single peptides or peptide mixes followed by ICS or are stained directly by MHC-multimers.

**Fig 3:**
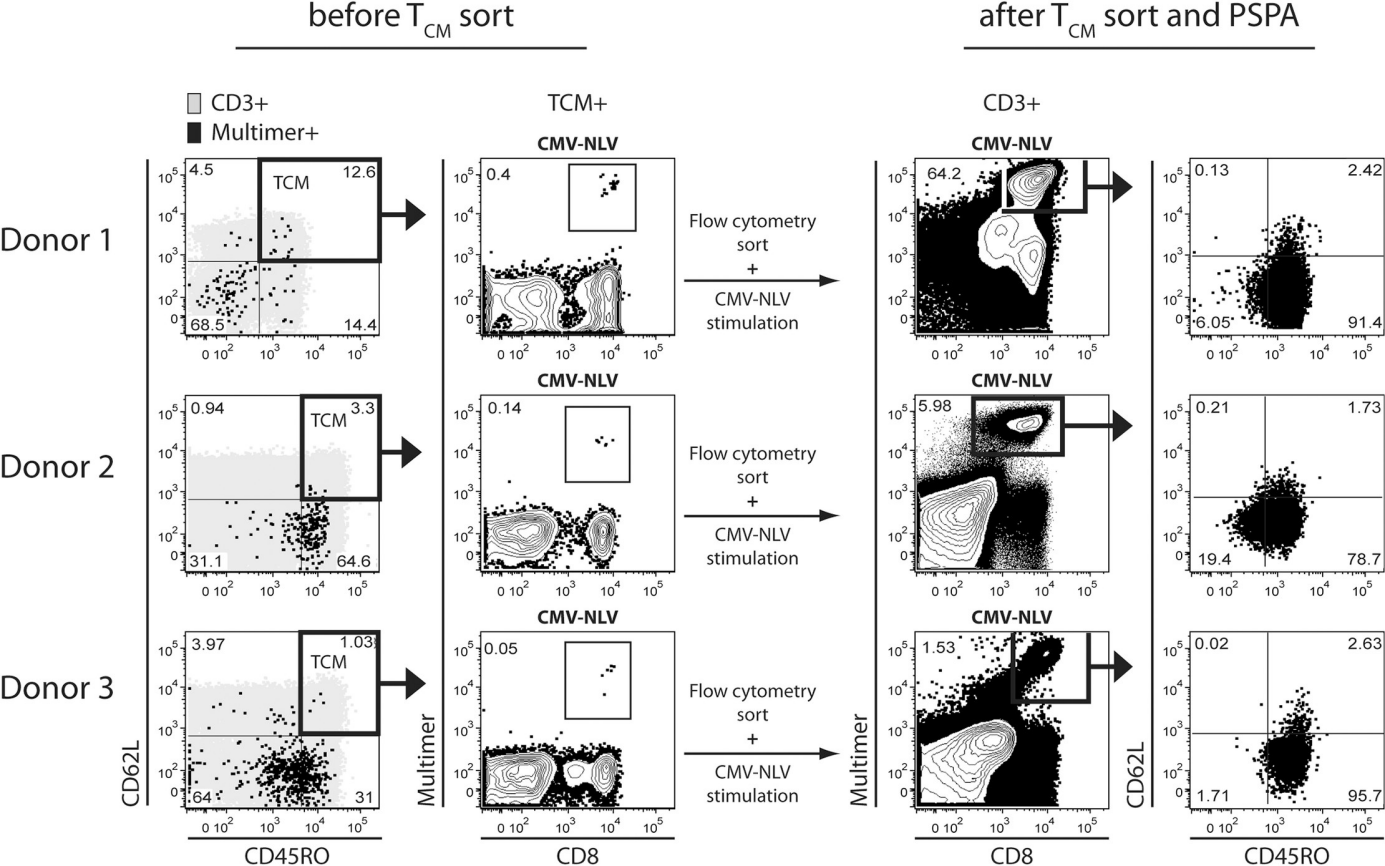
Minimal numbers of virus epitope-specific T_CM_ undergo extensive proliferation and phenotype differentiation. Human PBMC-derived CD3^+^ T cells of three healthy donors (Donor 1–3) were enriched using magnetic CD3-Fab-Streptamers. T_CM_ were subsequently isolated by FACS-sorting using anti-CD45RO and -CD62L fluorescent antibodies. Finally, isolated T_CM_ underwent a PSPA using CMV pp65-based HLA-A*02:01/NLV peptide. Ex-vivo-staining of PBMCs before enrichment and sorting is shown (before T_CM_ sort). CD3^+^ (grey) and CMV MHC-multimer^+^ CD8^+^ T cells (black) were analyzed with regard to their T_N_ (CD45RO^-^/CD62L^+^), T_CM_ (CD45RO^+^/CD62L^+^), T_EM_ (CD45RO^+^/CD62L^-^) and T_EMRA_ (CD45RO^-^/CD62L^-^) phenotype (far left column). Relative size of MHC-multimer^+^ T_CM_ is depicted (middle left column). After FACS-sorting and long-time CMV peptide stimulation (after T_CM_ sort and PSPA), cultured T_CM_ were analyzed regarding MHC-multimer^+^ CD8^+^ T cell frequency (middle right column) and phenotype differentiation (far right column).

As a next step we wanted to determine if the rare human CMV-specific T_CM_ share similar characteristics as their potent murine counterparts regarding proliferation and differentiation capacity [19]. We established a peptide-specific proliferation assay (PSPA) using autologous stimulator PBMC pulsed with either single peptides or peptide pools to enable the prolonged stimulation of single- or multi-epitope-specific T cell populations within the T_CM_ compartment (Fig 2). We sorted CD3^+^ T cells with a T_CM_ phenotype (CD45RO^+^ CD62L^+^) from three representative HLA-A*02:01^+^ PBMC donors with an ex-vivo-detectable CMV-NLV-specific CD8^+^ T cell population (Fig 3, before T_CM_ sort). As exemplarily found in Fig 1, T_CM_ frequencies among CMV-specific IFN-γ^+^ T cells after CMV-NLV peptide or CMV-pp65 and CMV-IE-1 peptide mix restimulation were low (S1 Fig). We subsequently stimulated the sorted CD3^+^ T cells with CMV-NLV single peptides. All donor T_CM_ cultures showed strong proliferation of the CMV NLV-specific CD8^+^ T cell population (Fig 3, after sort, left column). Starting from 0.05% (donor 3) - 0.4% (donor 1), equivalent to 125–1000 CMV epitope-specific T cells (Fig 3, before sort, right column), CMV-MHC-multimer^+^ CD8^+^ T_CM_ reached frequencies of 1.5% (donor 3) - 64% (donor 1) after sort and CMV peptide-specific stimulation (Fig 3, after T_CM_ sort and PSPA), which corresponds to a 30-160-fold increase of the epitope-specific population within the T_CM_ compartment. Moreover, T_CM_-derived CMV-NLV-specific CD8^+^ T cells developed into more differentiated phenotypes (mostly T_EM_) while preserving consistently a small T_CM_ population. Taken together, our results were in line with earlier observations of T_CM_ plasticity in animal models. Minimal numbers (125–1000 cells) of human epitope-specific T_CM_ could undergo extensive proliferation and matured into more differentiated T cell subsets.

### Clinical GMP-grade T_CM_ products contain a plethora of virus-specific T cells

To pursue the question if T_CM_ could potentially serve for therapeutic application, we investigated if the identified characteristics can be also observed in GMP-compatible clinical cell products.

**Fig 4:**
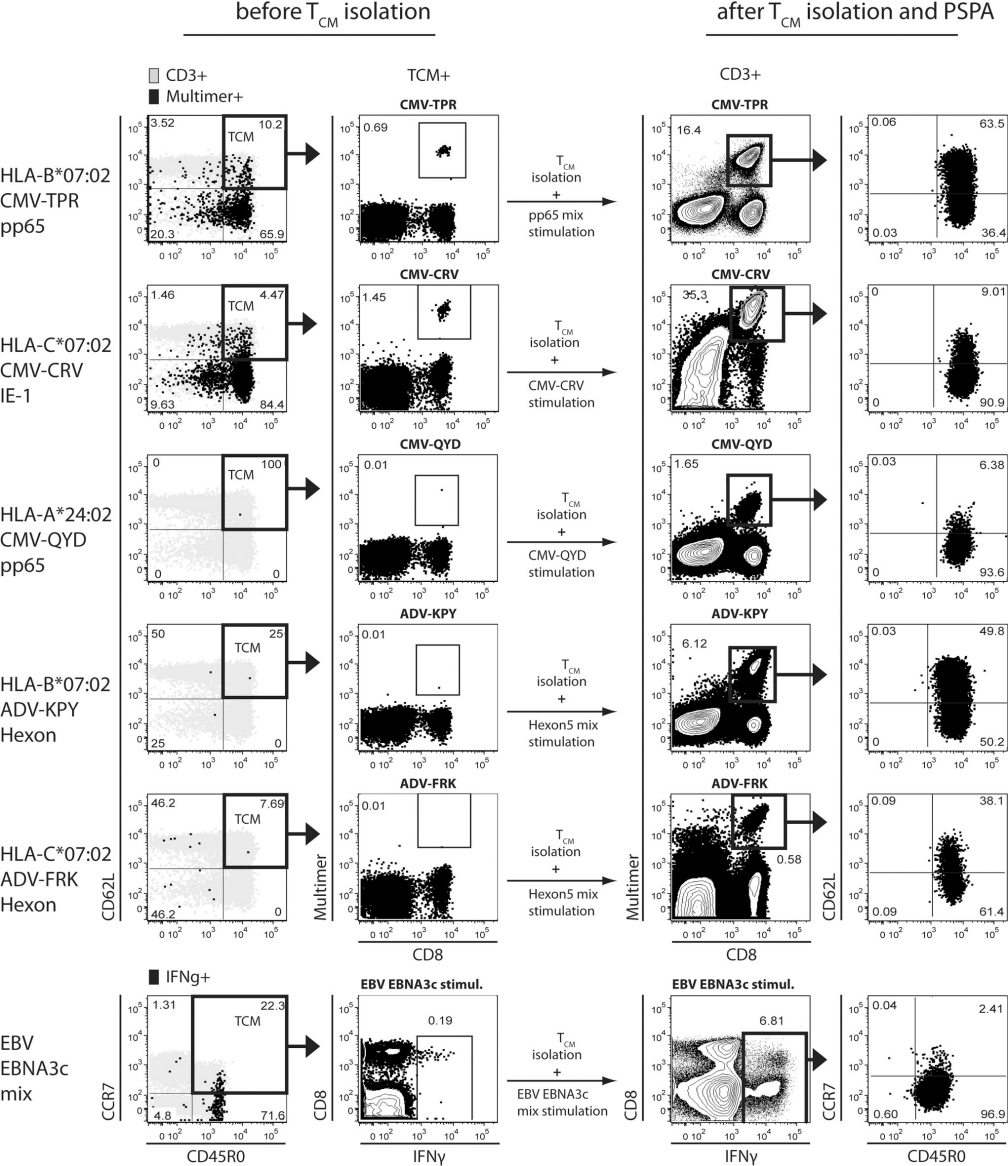
PSPA of GMP-grade T_CM_ products enables visualization of multiple, *ex vivo* undetectable T cell populations against various viruses. One representative non-mobilized leukapheresis product from a healthy donor is depicted. After performed leukapheresis, T_CM_ were generated the following day in a GMP-compatible process via two-step positive enrichment using CD3-Fab-Streptamers and CD62L-Fab-Streptamers, respectively, followed by a depletion with CD45RA-Fab-Streptamers. Subsequently, isolated T_CM_ underwent a PSPA using AdV Hexon-based and CMV pp65- and IE-1-based single peptides with restriction to HLA-B*07:02, -C*07:02 and A*24:02, as well as EBV EBNA3c peptide pool. Ex-vivo-staining of original donor PBMCs is shown (before T_CM_ isolation). CD3^+^ (grey) and virus MHC-multimer^+^ CD8^+^ T cells (black) or IFNγ^+^ CD8^+^ T cells (black, bottom row) were analyzed with regard to their T_N_, T_CM_, T_EM_ and T_EMRA_ phenotype (far left column). Relative sizes of MHC-multimer^+^ CD8^+^ T_CM_ or IFNγ^+^ CD8^+^ T_CM_ (bottom row) are depicted (middle left column). After T_CM_ isolation and long-time peptide stimulation (after T_CM_ isolation and PSPA), cultured T_CM_ were analyzed regarding MHC-multimer^+^ and IFNγ^+^ (bottom row) CD8^+^ T cell populations (middle right column) and phenotypical differentiation (far right column).

Three healthy volunteers were recruited to undergo leukapheresis for PBMC donation. Apheresis material was subsequently used to manufacture T_CM_-enriched cell (T_CM_) products (CD3^+^/CD62L^+^/CD45RA^-^) in a GMP manufacturing facility (TUMCells, Munich, Germany) using the recently introduced Fab-Streptamers [38]. In Fig 4 we exemplarily depicted one donor with a positive virus serology for AdV, CMV and EBV. This donor was initially screened for virus-specific T cell populations using MHC-multimer-staining and ICS according his confirmed HLA-Type (HLA-A*24:02, -A*31:01, -B*07:02, -B*51:01, -C*07:02, -C*15:02). T_CM_ frequencies among CMV-specific IFN-γ^+^ T cells after CMV-pp65 or CMV-IE-1 peptide mix restimulation were low (S2 Fig). Only two out of three CMV MHC-multimer^+^ populations available for the donor’s HLA-type could be detected *ex vivo*, a pp65-based B*0702-restricted TPR- and a recently described [47] IE1-based C*0702-restricted CRV population (Fig 4, before T_CM_ sort). For the AdV-specific B*0702-restricted KPY [40] peptide and the recently identified C*0702-restriced FRK [41] AdV epitope, no MHC-multimer^+^ populations could be detected. Finally, for EBV no suitable MHC-multimers were available. After PSPA, however, the presence of CD8^+^ T cells with specificity for every tested AdV- and CMV-based MHC-multimer could be unveiled in the T_CM_–product (Fig 4., after T_CM_ sort and PSPA). Virus-specific CD8^+^ T cells showed a 24- (CMV-TPR- and CRV-specific CD8^+^ T cells) to 612-fold (AdV-KPY-specific CD8^+^ T cells) increase in frequency with regard to T_CM_-derived CD3^+^ T cells.

Beside both AdV-specific CD8^+^ T cell populations, also an A*2402 CMV-QYD CD8^+^ T cell population became detectable emphasizing that the sensitivity advantage of PSPA was not restricted to a single virus. Furthermore, we detected functional EBV-specific CD3^+^ T cells expressing IFNγ upon short-term stimulation with EBNA3c peptide pool. While mostly CD8^+^ EBV-specific T cells were expressing IFNγ in ICS prior to PSPA, EBNA3c-specific CD4^+^ T cells dominated after PSPA. Finally, large populations of functional IFNγ^+^ CMV- and AdV-specific T cells were also detectable in PSPA upon stimulation with CMV pp65 and AdV hexon peptide mixes (S3 Fig). All but one (B*0702 CMV-TPR pp65) of the virus-specific T_CM_ cultures showed a differentiation predominantly into T_EM_, but conserved simultaneously in a minor fraction their T_CM_ phenotype (Fig 4). To further examine the functionality of proliferating T_CM_ after stimulation by PSPA, we used in an additional experiment another non-mobilized apheresis product from a healthy donor and generated a Fab-Streptamer-selected clinical grade T_CM_ product (S4 Fig). This time, we analyzed the functional state of proliferating T cells in more detail by doing ICS with IFN-gamma and TNF to look for polyfunctionality of virus-specific T cells. A substantial part of the strongly proliferating A2 CMV-pp65- and A2 EBV BMLF-1 -specific T cells was IFNg and TNF positive confirming their high functionality (S4A Fig). We used this same clinical grade T_CM_ product to look also for the expression of available co-inhibitory markers (PD-1, TIM-3 and LAG-3) on proliferated virus-specific T cells. Those markers can be sign of T cell exhaustion after continuous antigen stimulation, while In particular PD-1 expression can be also influenced by the activation and differentiation state of the analyzed T cells [48,49]. We found an intermediate PD-1 expression on roughly 50% of CMV- and EBV-peptide-specific CD8^+^ T cells after proliferation in PSPA. Low expression of LAG-3 was detectable on MHC multimer positive, virus-epitope-specific T cells, while TIM-3 was not detectable (S4B Fig). However, expression of LAG-3 and PD-1 on multimer-negative cells was more pronounced, including a substantial proportion of PD-1^hi^ T cells. Taken together, T_CM_-derived virus peptide-specific T cells after proliferation in PSPA show high proliferative capacity, polyfunctionality, intermediate expression of PD-1 and inconsistent expression of other co-inhibitory molecules.

Obviously, the up-scaled Fab-Streptamer-based GMP manufacturing of the T_CM_ product from standard leukapheresis material did not influence the high reconstitutional capacity of T_CM_.

### Functional virus-specific T_CM_ can be generated from a mobilized stem cell product

**Fig 5:**
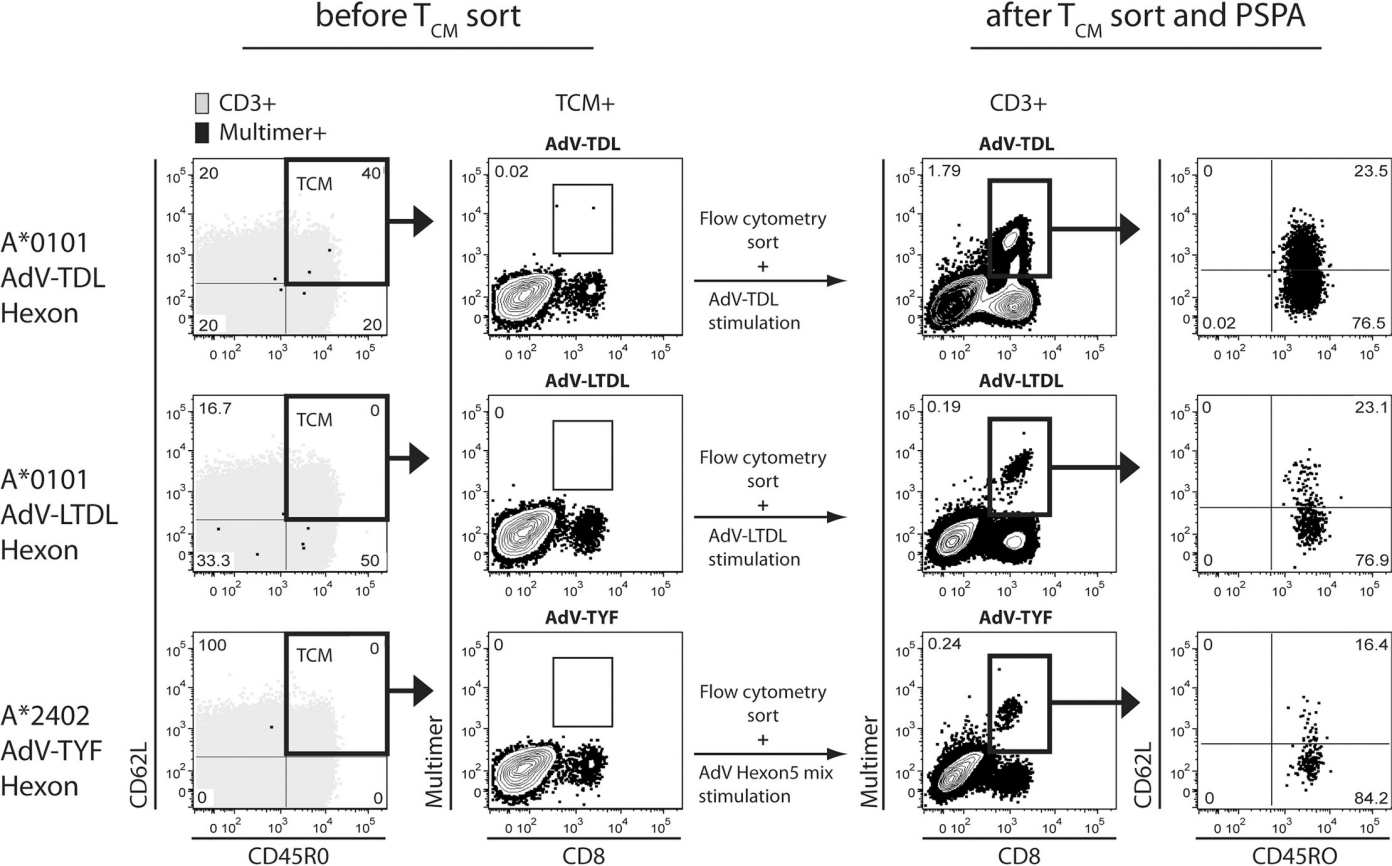
Virus epitope-specific T_CM_ can be generated from mobilized stem cell apheresis material. Highly pure (>95%) human mobilized stem cell apheresis-derived T_CM_ was generated by FACS-sorting using anti-CD3, -CD45RO and–CCR7 fluorescent antibodies. Subsequently isolated T_CM_ underwent a PSPA using AdV hexon-based HLA-A*01:01/TDL and HLA-A*01:01/LTDL peptides as well as AdV hexon 5 peptide pool (A*24:02/TYF population). Ex-vivo-staining of unsorted donor PBMC is shown (before T_CM_ sort). CD3^+^ (grey) and AdV MHC-multimer^+^ CD8^+^ T cells (black) were analyzed with regard to their T_N_, T_CM_, T_EM_ and T_EMRA_ phenotype (far left column). Relative size of MHC-multimer^+^ T_CM_ is depicted (middle left column). After FACS-sorting and long-time AdV-peptide stimulation (after T_CM_ sort and PSPA), cultured T_CM_ were analyzed regarding MHC-multimer^+^ CD8^+^ T cell frequencies (middle right column) and phenotypical differentiation (far right column).

As advantageous T_CM_ characteristics were maintained in GMP-conform T_CM_ products, we considered T_CM_ as suitable candidates for clinical application. T cells for adoptive transfer are mostly provided by the patient´s HLA-matched stem cell donor and lead to most beneficial results in this setting [29]. For logistic reasons and donor protection, T_CM_-products would be ideally generated in the alloHSCT setting from the mobilized stem cell apheresis directly after purification of CD34^+^ stem cells. This CD34-negative fraction contains vast amounts of functional PBMC. However, apheresis material from stem cell donors, who are mobilized with G-CSF to augment circulating CD34+ stem cells, differs (e.g. in cellular composition) from non-mobilized leukapheresis products. As these differences can potentially influence the envisioned ACT-products [37,50] we wanted to prove that functional T_CM_ can be conserved in mobilized stem cell material. Despite limited access due to medical and regulatory restrictions, we were able to receive a leftover frozen mobilized apheresis sample (Fig 5). Due to the limited cell numbers available, we generated T_CM_ by FACS-sorting (purity > 95%). AdV-specific T cells (donor was seronegative for CMV and EBV but seropositive for AdV) could not be identified during ex-vivo-staining with available donor-HLA-matching MHC-multimers. Following stimulation with single peptides (AdV HLA-A*01:01-restricted TDL and–LTDL) or peptide mixes (AdV HLA-A*24:02-restricted TYF) in PSPA, AdV hexon-based T cell populations became detectable (Fig 5, after T_CM_ sort and PSPA). Furthermore, AdV-specific T cells underwent phenotype differentiation mostly into T_EM_ and were functional with respect to IFNγ expression (S5 Fig). As multiple, tiny virus-specific T_CM_ populations could be made detectable by PSPA even in a (cryopreserved) mobilized stem cell apheresis sample, we conclude that this material can potentially serve as starting material for T_CM_-products.

## Discussion

For several advanced hematologic malignancies, especially AML, alloHSCT can be the only curative treatment option and despite persisting high mortality rates number of applications rise continuously [1,51–53]. Recently–due to the introduction of reduced intensity conditioning (RIC) regimes [54,55]–the indication for alloHSCT has been further expanded towards elderly patients reflecting the epidemiologic need for a disease that is diagnosed at a median age of 65 years [56,57]. At the same time, elderly patients are especially susceptible for (chronic) GVHD and infection, together representing major causes for alloHSCT-related mortality in this patient group. Several regimens for prevention of GVHD are under investigation and the depletion of T cells from the stem cell graft is one considered option [3]. At the same time, approaches focusing on the beneficial effects of T cells in the context of alloHSCT–namely protection against infections–receive a major attention. Several clinical studies have shown the curative potential of ACT and this safe treatment option has become more and more established in the past decade [15–20,22,23,58]. However, the huge variety of ACT approaches that are currently in use face specific challenges like the limited spectrum of covered pathogens, the time-race to provide “ready-to-use” T cell products, regulatory barriers and last but not least costs. With regard to the expected broad application of ACT in the future, these challenges become even more prominent.

Several recent data from animal models emphasized the potential of T_CM_ in this regard. It has been shown that this compartment has strong proliferative capacity, high reconstitution potential and can be protective at low cell numbers[19,32–35]. Furthermore, as compared to their T_N_ counterparts, they have at the same time a low allo-reactive potential [59–62].

An alloHSCT-setting using a T cell-depleted graft for GVHD prevention in combination with an early low dose transfer of a T cell subset for protective and safe immune reconstitution would address several challenges simultaneously and potentially lower all-cause mortality [3]. We previously showed that minimal numbers of T cells are able to induce clearance of CMV viremia in patients post alloHSCT [19]. However, the phenotype of those protective progenies is unknown and studies investigating explicitly the potential of human T_CM_ are missing to date. Our findings that in-vitro-stimulated human T_CM_ share characteristics with T_CM_ from animal models are in this context important. Moreover, we could show that GMP-conform T_CM_ products contain a plethora of functional and highly proliferative virus-specific T cells that undergo phenotype differentiation upon stimuli with different virus peptides. We concluded that transfer of human T_CM_ could be good approach for prophylactic broad spectrum ACT in patients with T cell-depleted alloHSCT and investigated its potential for a clinical trial setting.

CMV peptide-specific T_CM_ derived from buffy coats of healthy donors showed strong proliferation upon long-term single peptide stimulus. Moreover, these populations exhibited differentiation preferentially into T_EM_ and to a minor extent into late-differentiated T_EMRA_ showing similarities to past observations made in animal models [32]. Our developed PSPA played an essential role in this context as this assay simulates the challenge that a virus infection has on transferred T cells. We applied a number of only 2.5 x 10^5^ T_CM_ in the PSPA in order to mimic a low dose transfer of T cells. This corresponds to only 3500 cells per kg bodyweight in a 70kg patient, which was shown previously by our group to be curative in post-alloHSCT patients suffering from drug-resistant CMV reactivation [19]. However, in contrast to potential future clinical applications, T_CM_ in these initial experiments were still gained from peripheral blood and were generated partially by FACS-sorting.

Therefore, we generated GMP-conform CD3^+^/CD62L^+^/CD45RA^-^ T_CM_-enriched products derived from leukapheresis material of healthy volunteers using recently introduced clinical grade Fab Streptamers [38]. In order mimic the clinical setting as close as possible, donors were tested serologically for AdV, CMV and EBV and underwent high-resolution HLA-typing. Ex-vivo-screening for virus-specific T cells could thereby be matched prior to PSPA. The demonstrated T_CM_ product contained virus-specific T cells against all three viruses. The favorable HLA-type, for which multiple immunodominant epitopes are described, enabled us to use several MHC-multimers for broad spectrum diagnostics. We could show epitope-specific T cell populations for different HLA-alleles and viruses with strong proliferation and signs of differentiation. Moreover, virus-specific T cells were functional after PSPA with strong IFNγ expression upon CMV, AdV and EBV peptide mix stimulation. Beside the verification of the high proliferative capacity of virus-specific T_CM_ in the GMP-product, the PSPA helped us to identify additional antiviral T cell specificities within the T_CM_ compartment. Two AdV epitope-specific CD8^+^ T cell populations [40,41,63] that had been undetectable in the pre-PSPA T cell screening became visible after stimulation. The frequency of AdV-specific T cells in the peripheral blood is generally low [63] and is dominated by CD4^+^ T cells as depicted by ICS before PSPA. In this regard, the PSPA could become a valuable diagnostic tool to amplify the sensitivity for rare specificities within the T_CM_ compartment. This could also be true for underrepresented CMV-specific T_CM_ as illustrated by the detection of HLA-A*24:02-restricted CMV-QYD-specific T cells.

With an original frequency below the detection limit (0.01%) in ex-vivo stainings, some virus-specific T_CM_ specificities consisted of 25 cells or less in our assay. Hence, their rise to substantial frequencies in PSPA proved their strong proliferative capacity. Together with the observed IFNγ expression after proliferation they fulfilled two important requirements for clinical virus control after ACT, proliferation and functionality [64]. In the more detailed functional analysis of a second Fab-Streptamer-selected clinical grade TCM product polyfunctionality of proliferating T cells was indicated by substantial and comparable expression of both IFN-γ and TNF. Analysis of the expression of co-inhibitory markers (PD-1, TIM-3 and LAG-3), which has been described for exhausted virus- and tumor-secific T cells, was less conclusive after proliferation in PSPA. Intermediate PD-1 expression on a part of CMV- and EBV-peptide-specific CD8^+^ T cells, low expression of LAG-3 and absent TIM-3 expression was found on MHC multimer positive, virus-epitope-specific T cells. PD-1 ^hi^ T cells, which are associated with an irreversible dysfunctional state [65], were exclusively found on MHC multimer-negative T cells. In particular expression of PD-1 could be also influenced by the activation and differentiation state of the analyzed T cells [48,49]. Activated T cells can transiently express PD-1, expression on intermediate differentiation states (T_EM_) of virus-specific CD8^+^ T cells was also found [66,67]. Taken together, we do not regard it as likely that virus peptide-specific T cells after proliferation in PSPA show true signs of exhaustion. However, we cannot exclude that, under the chosen conditions of strong and repetitive in vitro stimulation, which allows very efficient detection of minute amounts of virus-specific T cells in PSPA, a part of the strongly expanding T cells eventually upregulate coinhibitory receptors. To study this in more detail in the future, the recently described exhaustion-associated transcription factor TOX [65], which is correlated with high expression of PD-1 and other coinhibitory receptors on antigen-experienced T cells [68], could be an interesting marker to study the functional state of proliferating virus-specific T cells, particularly after in vivo transfer in current running clinical trials.

In consequence, we consider that the selection of protective T cell products primarily based on the T_CM_ phenotype is a feasible approach. Furthermore, despite the limitations of an in-vitro assay, these results for human T_CM_ were in line with our previous observations that protection against systemic infections can be achieved by transfer of minimal T cell numbers or even single cells [19,32,33].

However, to allow the expansion and differentiation of low doses of T_CM_ competing with virus replication in immunocompromised individuals, this application would be best suited for an early, prophylactic transfer. Since the T_CM_ compartment contains presumably progenies against all T cell-controlled pathogens an individual had contact with before, a prophylactic product from al healthy donor would cover a broad spectrum of pathogens. Due to limited knowledge of immunodominant epitopes for different HLA-types and viruses, many of those specificities included in a T_CM_-product would not be attainable with conventional MHC-multimers. Additional advantages of this new approach are the coverage of both CD8^+^ and CD4^+^ T cells and its logistical simplicity without requirement for virus-specific T cell screens in seropositive donors before transfer. Furthermore, in comparison to the depletion of CD45RA [37,69], we can target a more defined T cell product (TCM), which could be made usable in the future also as a starting cell fraction for the generation of TCR- or CAR-engineered T cells.

These advantageous features of a broad-spectrum T_CM_ product come along with an initial ignorance of the comprised repertoire in comparison to defined MHC-Streptamer-isolated virus epitope-specific T cell transfers. This requires an extensive and detailed personalized immunological analysis of the T_CM_ product beforehand in order to predict protective capacity and enable targeted post-transfer T cell monitoring. The described PSPA will allow such a detailed analysis of a T_CM_ product’s spectrum. Virus-specific peptide mixes can be used for stimulation and analysis of T_CM_ as immunodominant epitopes will be not entirely known. Expanded T_CM_ will then be further analyzed using available MHC multimers, ICS and molecular biology (e.g. T cell receptor (CDR3) identification or chimerism analysis for later ex vivo donor/host T cell discrimination). Finally, PSPA can be also used for the sensitive detection of low levels of functional virus-specific T_CM_ after transfer in patient’s PBMCs. This would presumably indicate protection against future replication of the respective virus, a valuable prognostic information.

Isolation of T_CM_ from the mobilized stem cell apheresis after CD34 stem cell purification would be an elegant way for future prophylactic ACT treatment of alloHSCT recipients. Therefore, the PSPA results from the mobilized apheresis product with a strong proliferation of virus-specific T_CM_ are encouraging. However, generation of T_CM_-products from mobilized stem cell material using currently available Fab-Streptamers is still limited by technical and regulatory restrictions (e.g. CD62L-shedding). Until these barriers are eliminated, T_CM_-products can be generated from a separate non-mobilized leukapheresis product. Nevertheless, mobilized apheresis remnants after CD34^+^ stem cell enrichment can be very useful in a clinical trial setting as a highly valuable PBMC source for donor T_CM_ compartment characterization by PSPA, allowing clinical efficacy testing with regard to pathogen-specific T cell expansions.

In summary, we consider that the reduction of GVHD via depletion of alloreactive T cells from the stem cell graft and a subsequent prophylactic low dose transfer of purified T_CM_ could become a new innovative approach and serve as a potent combination and ideally lower overall-mortality in alloHSCT. Previous data described T_CM_ as fulfilling important requirements for adoptive T cell prophylaxis and our data could confirm several of these features for human T_CM_. Beside an application at low numbers, data regarding the safety profile of transferred T_CM_ seems to be promising. Furthermore, T_CM_ qualify for broad application because the phenotype-based isolation is a uniform procedure with available GMP-compatible clinical grade Fab-streptamer reagents. In consequence, such a prophylactic stem cell donor-derived low dose transfer of T_CM_ is currently being tested in alloHSCT patients (PACT, Eudra-CT: 2015-001522-41) to determine its feasibility and safety. The PSPA described In this work was an essential component to demonstrate the potency of T_CM_ in vitro and receive clinical trial approval.

## Supporting information

S1 Fig Phenotype of the CMV-specific T cell repertoire after peptide mix restimulation. Ex-vivo-stainings of original PBMCs from the three donors (donor 1, 2 and 3) described in Fig 3 are shown. PBMCs were restimulated with either CMV-pp65 (upper left) and CMV-IE1 (lower left) peptide mix or with CMV pp65-based HLA-A*02:01/NLV peptide (upper right). Restimulated T cells were examined for antigen-specific IFNγ production. As a negative control, cells were stained without restimulation (no peptide, lower right). CD3^+^ (grey) and IFNγ^+^ CD3^+^ T cells (black) were analyzed with regard to their T_N_, T_CM_, T_EM_ and T_EMRA_ phenotype. Relative sizes of IFNγ^+^ CD3^+^ T cells are indicated for the four T cell subsets. For CMV-pp65 and CMV-IE-1 peptide-mix-restimulated T cells, the contribution of CD8^+^ (middle right column) and CD4^+^ T cells to the IFNγ^+^ CMV-specific T cell compartment are additionally depicted.
(PDF)

S2 Fig Phenotype of apheresis donor’s CMV-specific T cell repertoire after peptide mix restimulation. Ex-vivo-staining of original PBMCs from the donor described in Fig 4 is shown. PBMCs were restimulated either with CMV-pp65 (top row) or CMV-IE1 (bottom row) peptide mixes and examined for antigen-specific IFNγ production (far left column). CD3^+^ (grey) and IFNγ^+^ CD3^+^ T cells (black) were analyzed with regard to their T_N_, T_CM_, T_EM_ and T_EMRA_ phenotype (middle left column). Relative sizes of IFNγ^+^ CD3^+^ T cells are indicated for the four T cell subsets. The contribution of CD8^+^ (middle right column) and CD4^+^ T cells to the IFNγ^+^ CMV-specific T cell compartment is depicted.
(PDF)

S3 Fig GMP-grade T_CM_ product-derived AdV- and CMV-specific T cells express IFNγ. Isolated T_CM_ from the donor described in Fig 4 underwent a PSPA using CMV pp65 and AdV Hexon5 and Hexon3 peptide pool. ICS was performed with corresponding peptide pools in original donor PBMCs *ex vivo* (before T_CM_ isolation) and subsequently after T_CM_-enrichment followed by PSPA (after T_CM_ isolation and PSPA). Pregated on CD3^+^, CD8^+^ T cells were analyzed regarding IFNγ production.
(PDF)

S4 Fig Functionality of proliferating virus-specific T cells after PSPA of a GMP-grade T_CM_ product. An additional non-mobilized leukapheresis product from a healthy donor was used for generation of a clinical T_CM_ product in analogy to Fig 4. Fab-Streptamer-selected T_CM_ underwent a PSPA using HLA-A*02:02-restricted CMV pp65- (NLV) and EBV BMLF-1 (GLC)-based single peptide stimulation. On day 16 after stimulation, T cell cultures were analyzed for proliferation and functionality using ICS and MHC-multimers. **(A)** After CMV NLV (left) and EBV GLC (right) peptide restimulation, peptide-specific cytokine production of CD3+ T cells was analyzed in ICS. CD3/IFNγ and CD3/TNF stainings (gating: living lymphocytes) are shown. **(B)** CMV NLV- and CMV GLC- MHC multimers were used to stain virus peptide-specific T cells and their PD-1 (top row), LAG-3 (middle row) and TIM-3 (bottom row) expression was determined. As background controls, multimer stainings without the respective inhibitory marker staining (FMO) are shown. An exemplary plot for the gating strategy of living CD3^+^ T cells is demonstrated (top left).
(PDF)

S5 Fig AdV-specific T_CM_ maintain functionality in mobilized stem cell apheresis samples. Isolated T_CM_ from the donor described in Fig 5 underwent a PSPA using AdV Hexon5 peptide pool (33 days) and AdV hexon-based HLA-A*01:01/TDL and HLA-A*01:01/LTDL single peptides. ICS was performed with corresponding peptides in unsorted donor PBMCs *ex vivo* (before T_CM_ sort) and subsequently after T_CM_-enrichment and following PSPA (after T_CM_ sort and PSPA). Pregated on CD3^+^, CD8^+^ T cells were analyzed regarding IFNγ production.
(PDF)
