# Supplementary figures and images for: Targeted in-vitro-stimulation reveals highly proliferative multi-virus-specific human central memory T cells as candidates for prophylactic T cell therapy
Source: PLoS One. 2019 Sep 30;14(9):e0223258. doi: 10.1371/journal.pone.0223258 (PMC6768573; doi:10.1371/journal.pone.0223258)

Fig. S1

Donor 1

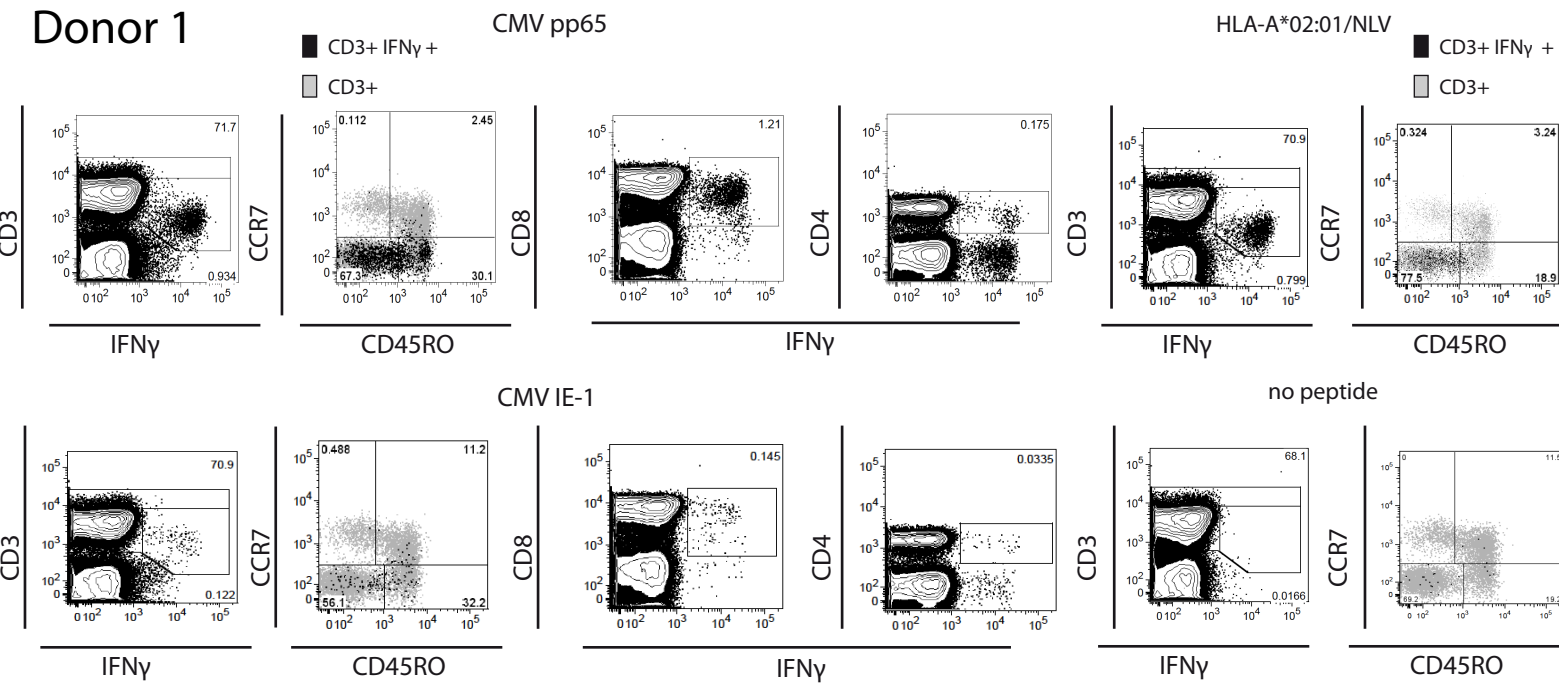

Donor 2

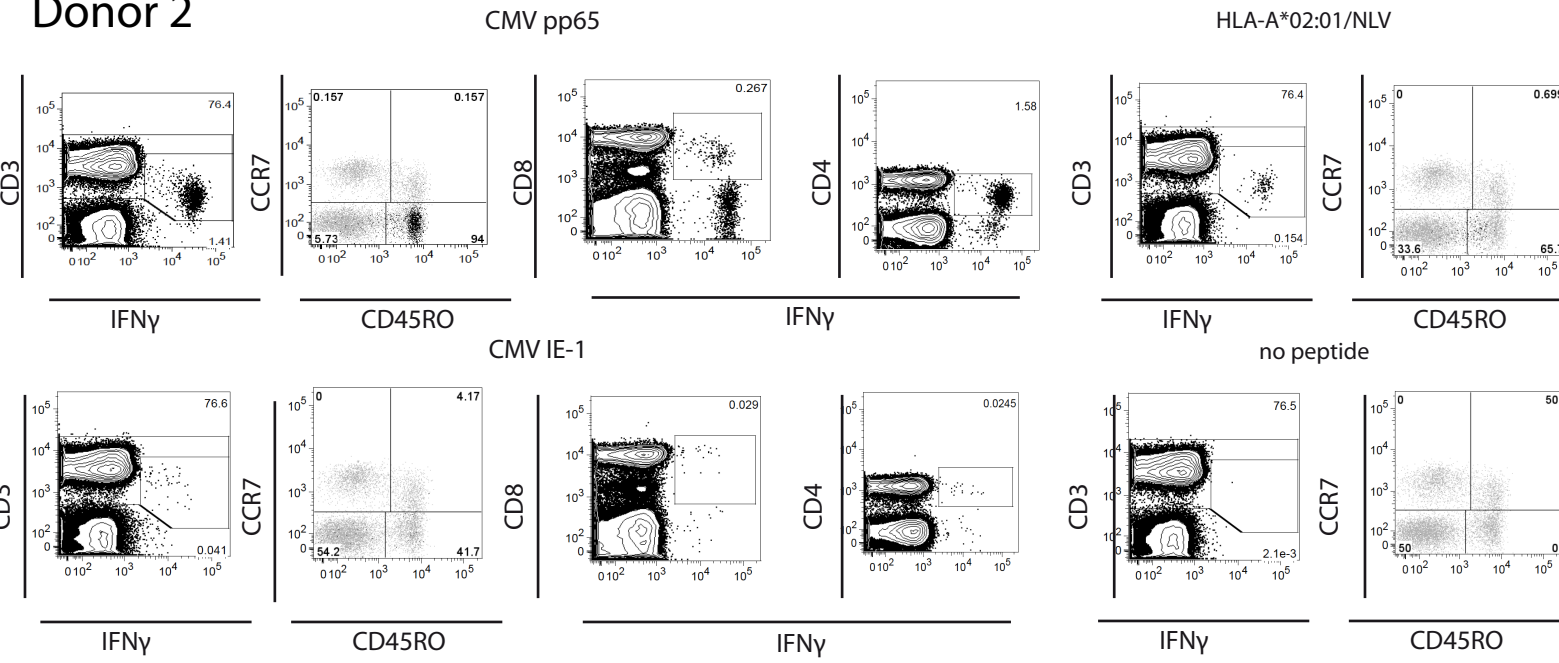

Donor 3

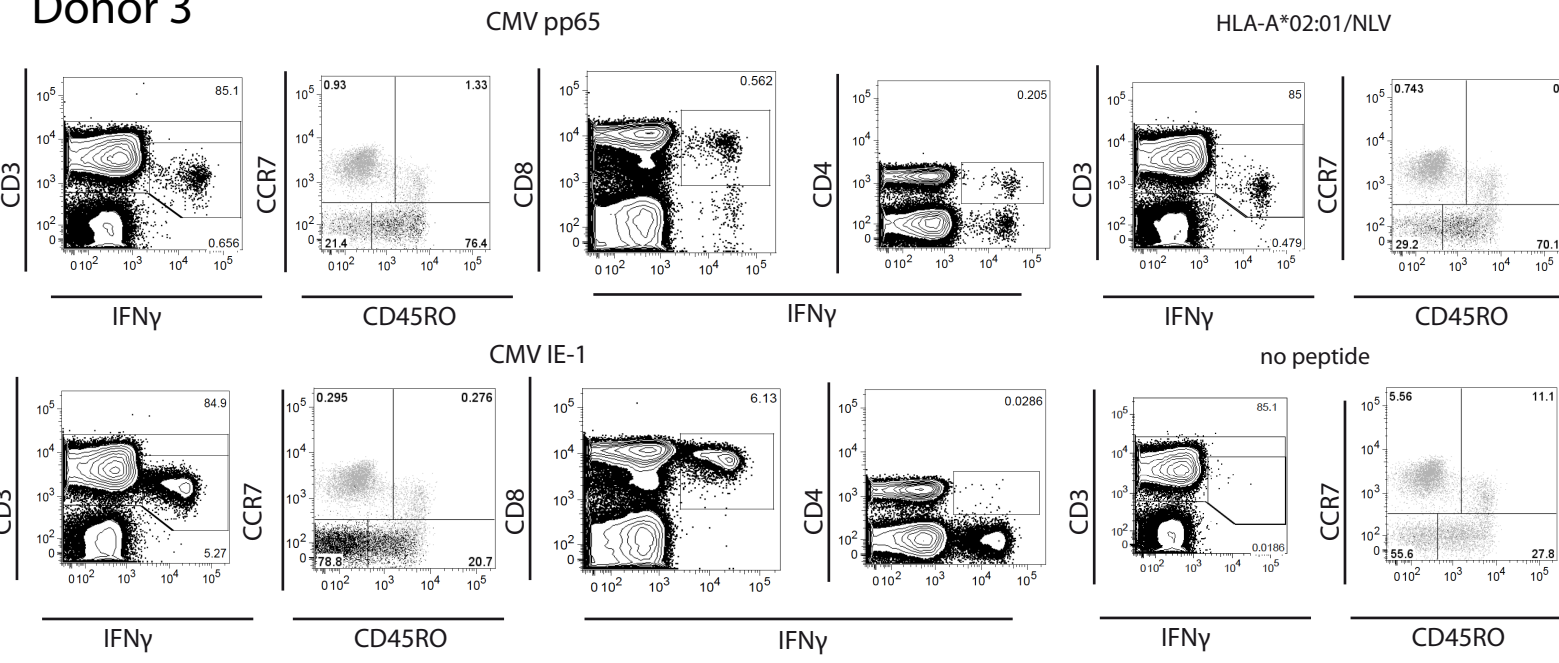

Supplement: S1 Fig — Ex-vivo-stainings of original PBMCs from the three donors (donor 1, 2 and 3) described in Fig 3 are shown. PBMCs were restimulated with either CMV-pp65 (upper left) and CMV-IE1 (lower left) peptide mix or with CMV pp65-based HLA-A*02:01/NLV peptide (upper right). Restimulated T cells were examined for antigen-specific IFNγ production. As a negative control, cells were stained without restimulation (no peptide, lower right). CD3+ (grey) and IFNγ+ CD3+ T cells (black) were analyzed with regard to their TN, TCM, TEM and TEMRA phenotype. Relative sizes of IFNγ+ CD3+ T cells are indicated for the four T cell subsets. For CMV-pp65 and CMV-IE-1 peptide-mix-restimulated T cells, the contribution of CD8+ (middle right column) and CD4+ T cells to the IFNγ+ CMV-specific T cell compartment are additionally depicted. (PDF) [file pone.0223258.s001.pdf]

Fig. S2

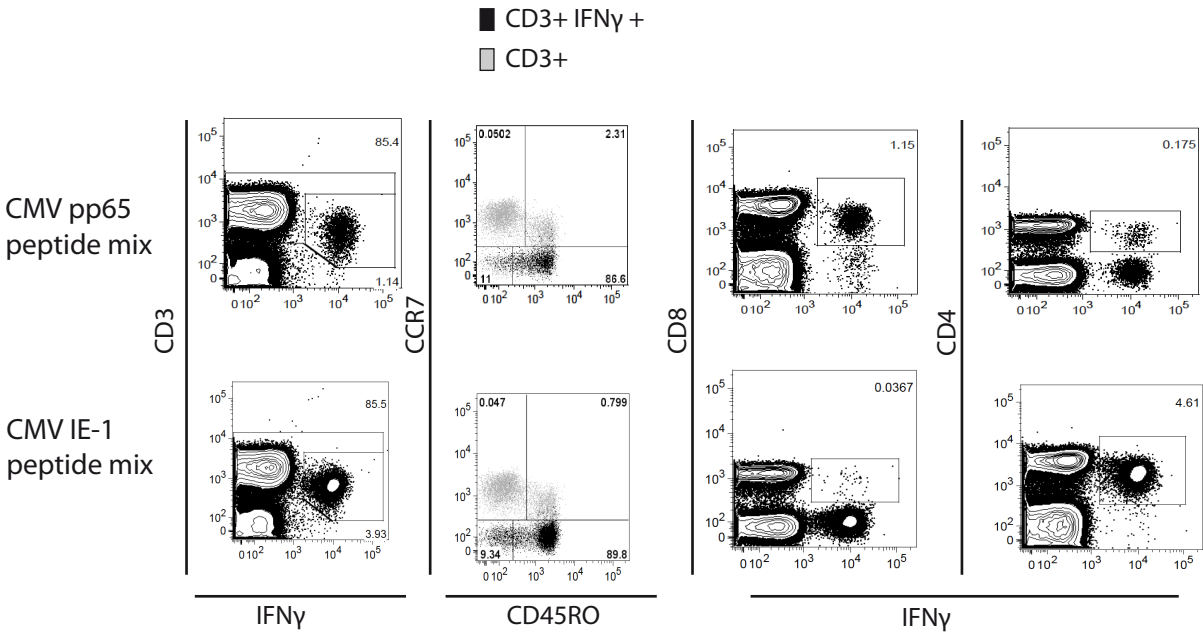

Supplement: S2 Fig — Ex-vivo-staining of original PBMCs from the donor described in Fig 4 is shown. PBMCs were restimulated either with CMV-pp65 (top row) or CMV-IE1 (bottom row) peptide mixes and examined for antigen-specific IFNγ production (far left column). CD3+ (grey) and IFNγ+ CD3+ T cells (black) were analyzed with regard to their TN, TCM, TEM and TEMRA phenotype (middle left column). Relative sizes of IFNγ+ CD3+ T cells are indicated for the four T cell subsets. The contribution of CD8+ (middle right column) and CD4+ T cells to the IFNγ+ CMV-specific T cell compartment is depicted. (PDF) [file pone.0223258.s002.pdf]

Fig. S3

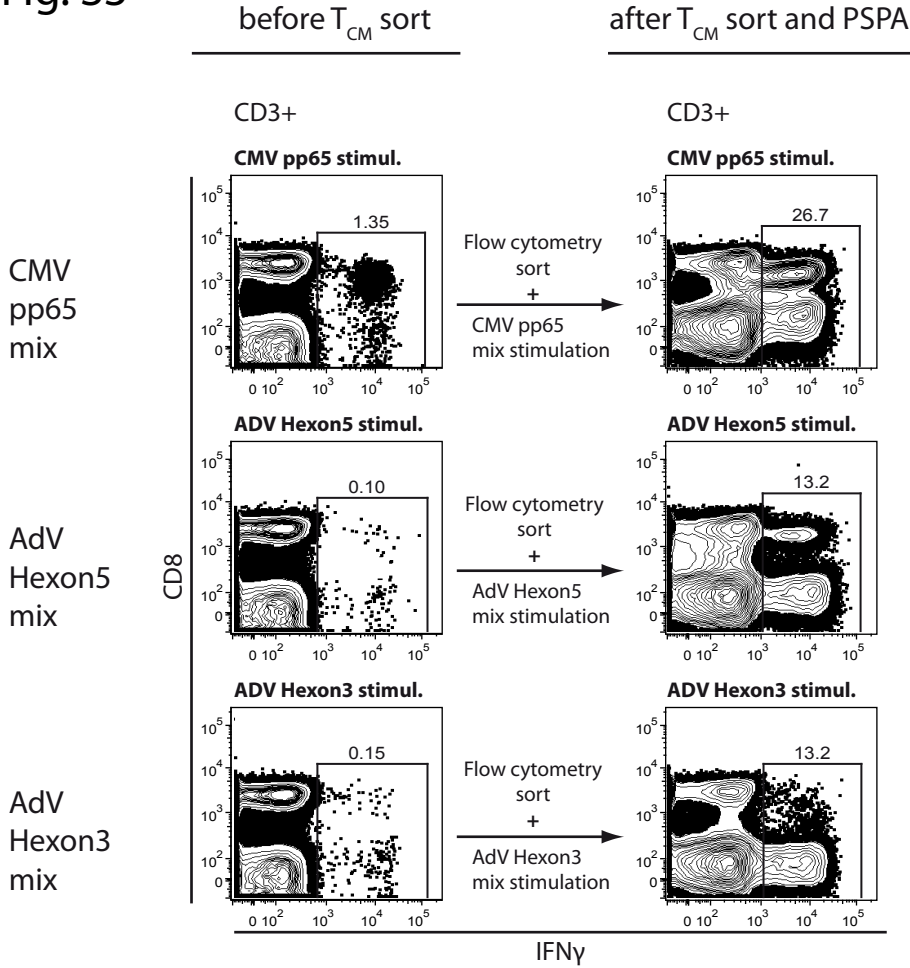

Supplement: S3 Fig — Isolated TCM from the donor described in Fig 4 underwent a PSPA using CMV pp65 and AdV Hexon5 and Hexon3 peptide pool. ICS was performed with corresponding peptide pools in original donor PBMCs ex vivo (before TCM isolation) and subsequently after TCM-enrichment followed by PSPA (after TCM isolation and PSPA). Pregated on CD3+, CD8+ T cells were analyzed regarding IFNγ production. (PDF) [file pone.0223258.s003.pdf]

Fig. S4

A)

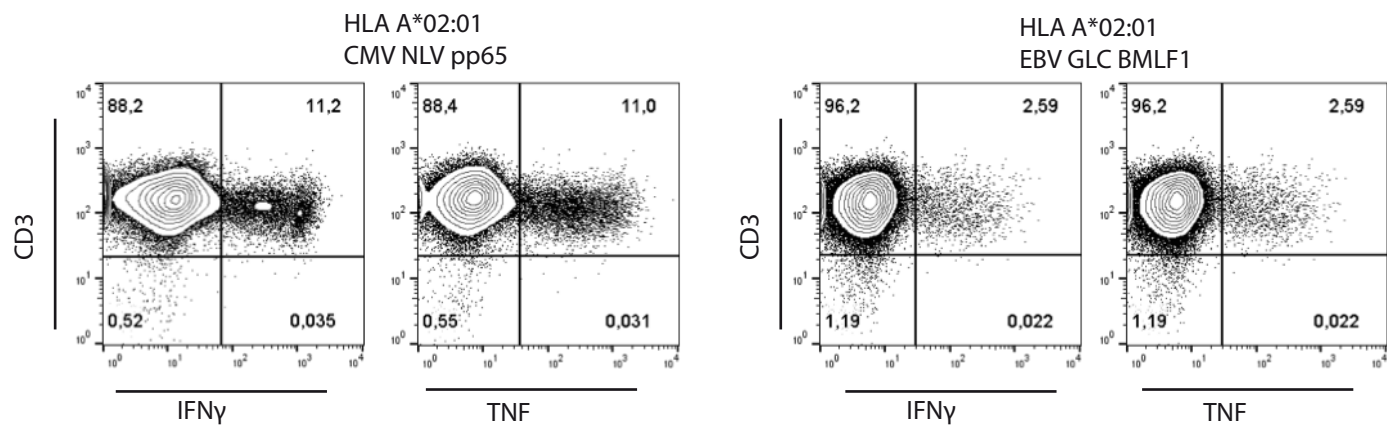

B)

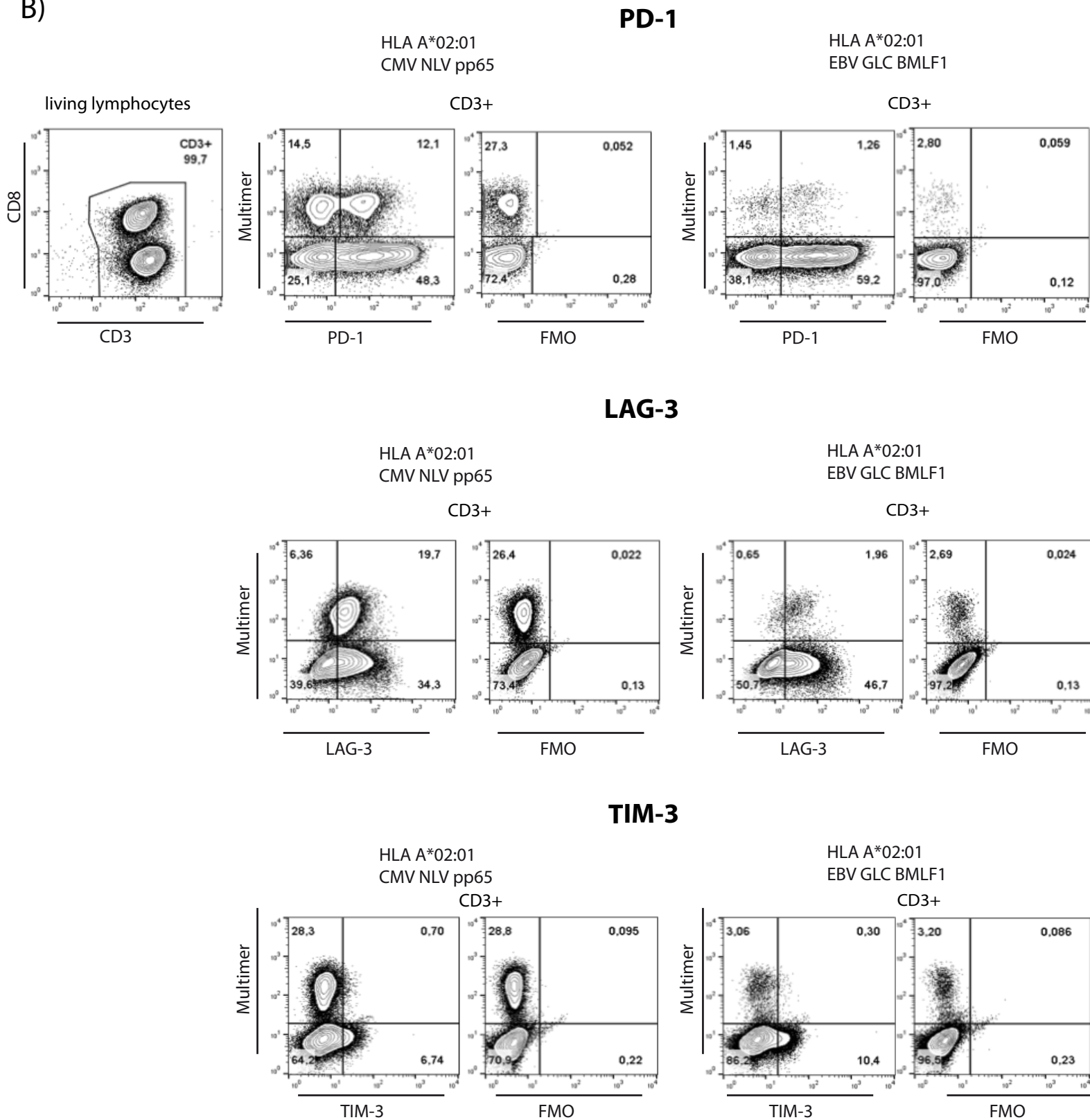

Supplement: S4 Fig — An additional non-mobilized leukapheresis product from a healthy donor was used for generation of a clinical TCM product in analogy to Fig 4. Fab-Streptamer-selected TCM underwent a PSPA using HLA-A*02:02-restricted CMV pp65- (NLV) and EBV BMLF-1 (GLC)-based single peptide stimulation. On day 16 after stimulation, T cell cultures were analyzed for proliferation and functionality using ICS and MHC-multimers. (A) After CMV NLV (left) and EBV GLC (right) peptide restimulation, peptide-specific cytokine production of CD3+ T cells was analyzed in ICS. CD3/IFNγ and CD3/TNF stainings (gating: living lymphocytes) are shown. (B) CMV NLV- and CMV GLC- MHC multimers were used to stain virus peptide-specific T cells and their PD-1 (top row), LAG-3 (middle row) and TIM-3 (bottom row) expression was determined. As background controls, multimer stainings without the respective inhibitory marker staining (FMO) are shown. An exemplary plot for the gating strategy of living CD3+ T cells is demonstrated (top left). (PDF) [file pone.0223258.s004.pdf]

Fig. S5

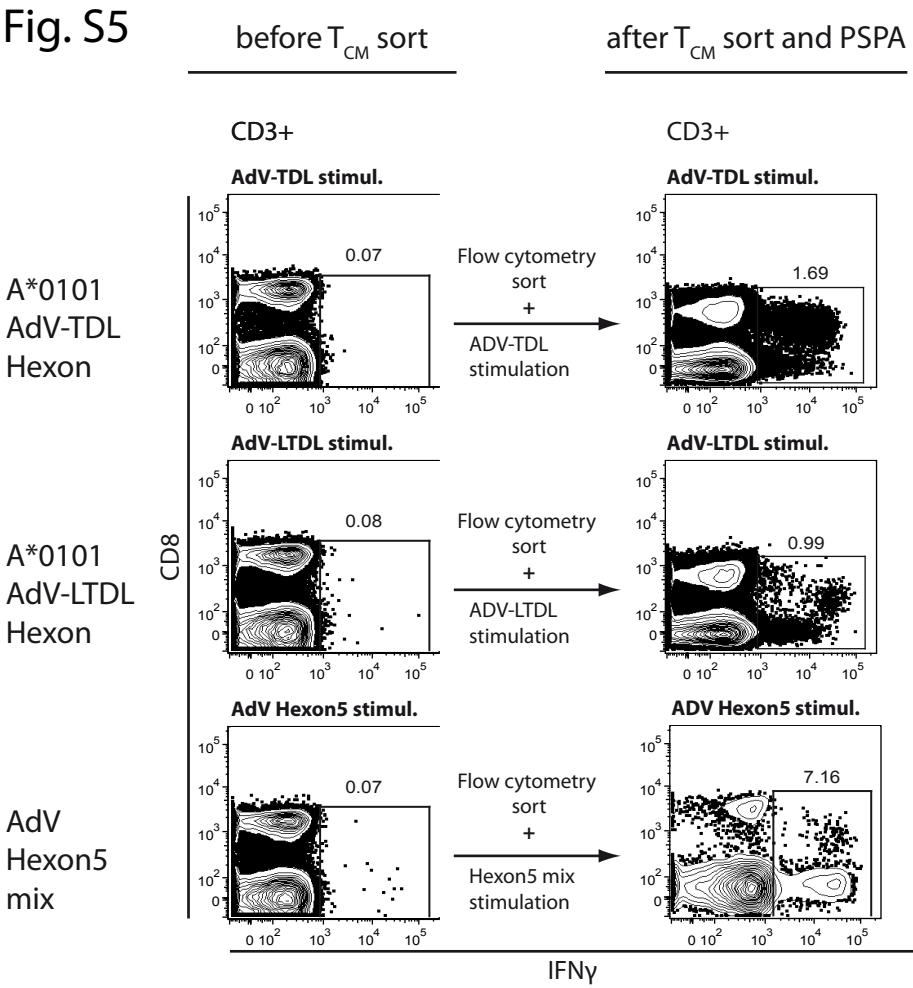

Supplement: S5 Fig — Isolated TCM from the donor described in Fig 5 underwent a PSPA using AdV Hexon5 peptide pool (33 days) and AdV hexon-based HLA-A*01:01/TDL and HLA-A*01:01/LTDL single peptides. ICS was performed with corresponding peptides in unsorted donor PBMCs ex vivo (before TCM sort) and subsequently after TCM-enrichment and following PSPA (after TCM sort and PSPA). Pregated on CD3+, CD8+ T cells were analyzed regarding IFNγ production. (PDF) [file pone.0223258.s005.pdf]
